# Long‐term patterns of urinary pyroglutamic acid in healthy humans

**DOI:** 10.14814/phy2.12706

**Published:** 2016-02-23

**Authors:** Richard S. Lord

**Affiliations:** ^1^ NGA BioscienceWaleskaGeorgia

**Keywords:** Pyroglutamic acid, 5‐oxoproline, glutathione, physiological cycles

## Abstract

An investigation of human biological variation in urinary organic acids, including pyroglutamic acid along with 39 other compounds, was previously reported in which levels were determined for 8 weeks in healthy adult subjects. Here, unique, 4‐week‐long physiological trends for one of those compounds, pyroglutamic acid (PGA), are reported. When PGA levels for an individual rose above 40 *μ*g/mg creatinine, 4‐week downward progressions occurred until levels reached values near 15 *μ*g/mg creatinine and the pattern was reversed when levels for an individual were below that level in the early weeks of the study. The pattern was especially prominent among 8 of the 13 menstruating female subjects suggesting a possible association with metabolic stress of the menstrual cycle. However, it also appeared in 3 of the 8 male subjects where other sources of metabolic stress may be present. The menstrual association is consistent with estrogen‐mediated increase in oxidative stress. Since PGA is linked to glutathione turnover, the consistency of extreme values across all individuals displaying the pattern indicates that 15 and 40 *μ*g/mg creatinine may represent limits that trigger shifts in sulfur amino acid metabolism. This is the first observation of approximate month‐long cyclic responses in a glutathione‐related urinary marker in humans.

## Introduction

Elevated urinary L‐pyroglutamic acid (PGA) has been reported as an index of glycine insufficiency since levels rise when glycine is insufficiently available to support glutathione recovery via the renal gamma‐glutamyl cycle (Jackson et al. 1987). The same group later demonstrated that, during pregnancy, urinary PGA rises much higher in Jamaican women compared to those in Southampton, UK, indicating differences in protein (and glycine) intake (Jackson et al. 1997). Further studies confirmed that restriction of glycine or sulfur amino acids alter pyroglutamate kinetics and urinary excretion (Metges et al. 2000). The studies were extended to reveal that severely burned adult humans have higher pyroglutamate clearance and compromised glutathione status (Yu et al. 2002). A series of case reports have linked PGA to acetaminophen‐induced high anion gap metabolic acidosis (Emmett 2014). Authors of these acetaminophen cases generally have agreed that the key metabolic linkage is the *γ*‐glutamyl cycle that operates to transport amino acids in the kidney, small intestines, and liver. The cycle normally consumes a large fraction of circulating glutathione that requires resynthesis from available precursors, glycine and cysteine. Thus, elevated urinary PGA indicates difficulty with maintenance of glutathione status whether the stress is experimental benzoic acid loading, dietary protein deficiency, or depletion of glutathione by detoxification pathways of acetaminophen or other drugs.

## Methods and Results

**Figure 1:**
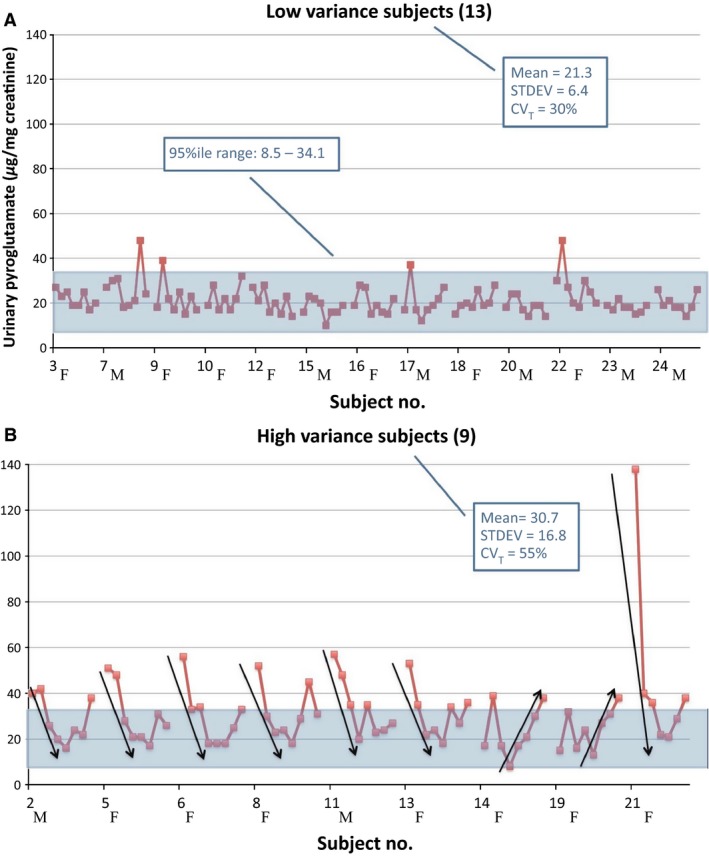
Sets of 8 weekly levels of urinary pyroglutamic acid for 22 subjects are shown divided into low‐variance (A) and high‐variance (B) groups as defined in the text. Gender is indicated beside subject numbers. Arrows were added to indicate patterns of four or more points shifting downward or upward.

Pyroglutamic acid (PGA or 5‐oxoproline) was measured in urine specimens submitted once weekly for 8 weeks by 22 subjects as part of an IRB‐approved study to determine biological variability in a profile of organic acids (Lord et al. 2012). The between‐run analytical variability for pyroglutamate was 3.5% at 50.9 μg/g creatinine, a value anomalously different from its calculated overall biological variability (CV_b_) of 33.5%, indicating that something other than analytical variability was contributing to the observed patterns. Figure 1 shows the 8‐week patterns found for all subjects divided into low‐variance (A) and high‐variance (B) sets. Unlike the other 39 other compounds included in that study, PGA levels in nine of the subjects demonstrated systematic trends over 4 or more weeks that contributed to its apparently high biological variability. Subjects with sequences of four or more downward‐ or upward‐shifting levels (indicated by arrows placed alongside the data points in Fig. 1) were initially defined as “high variance.” A 95 percentile range (8.5–30.1 *μ*g/mg creatinine) was derived from the remaining set of “low‐variance” subjects, and the criteria for high variability was restricted by requiring that two or more values during the 8 weeks fall outside of that range. This required subjects 17 and 22 to be moved from high‐ to low‐variance status, and CVs and reference range were recalculated. Most high variance subjects were female as shown by “M” or “F” next to subject numbers. Subject 11, the only male reporting decades of heavy cigarette smoking, subsequently died of lung cancer. When biological variability of PGA was calculated using only the 12 subjects who displayed no large, systematic shifts, coefficients of variability dropped from 33% to 23%.

## Discussion

The high‐variance patterns suggest 4‐week cycles of metabolic adaptation, and the preponderance of the occurrence in women may reflect the metabolic stress of the menstrual cycle. Other studies have established associations of plasma estradiol levels with factors related to oxidative stress such as plasma selenium (Ha and Smith 2003) and serum glutathione peroxidase (Darie et al. 1998). Direct measurements of adipose and breast tissue glutathione concentrations were also found to display synchronous variation with the menstrual cycle, with higher levels found in the mid‐luteal phase compared with follicular phase (Dabrosin et al. 2013). Since urinary PGA is a surrogate marker for status of glycine or sulfur amino acid reserves required for cysteinylglycine formation, a rate‐limiting step in the formation of glutathione (Persuad et al. 1996) and estrogen hormone actions stress glutathione responses, it is proposed that the female preponderance for strong pyroglutamate excursions is due to the oxidative stress enhancement of menstruation in these nominally healthy women. These data challenge a definition of “healthy” since improved glutathione status is achievable under appropriate nutritional conditions (Kent et al. 2003). Those women in the study who did not show large pyroglutamate excursions might be qualified by this criterion as healthy.

Since some men also displayed the 4‐week trends, and some women, like most of the men, displayed quite low study variance, the effect may depend on multiple factors that govern metabolic stability under conditions where oxidative stress or detoxification challenge cause increased glutathione demands. Urinary PGA levels above 40 *μ*g/mg creatinine appear to initiate 4‐week‐long decreasing PGA trends (see subjects 12 and 17), while levels below 15 *μ*g/mg creatinine (see subjects 3, 4 and 6) tend to initiate increasing trends that also persist for about 4 weeks or until levels rise to values near 40 *μ*g/mg creatinine. Both trending patterns resulted in PGA levels returning to the 15–40 *μ*g/mg creatinine range. Other than the typical month‐long menstruation cycles there is no reason to expect that other stresses would produce such long‐term trends. Plasma PGA (5‐oxoproline) changes during oral glucose tolerance testing were investigated as part of a metabolomics study to determine longitudinal changes in circulating metabolite patterns (Campbell et al. 2014). Diet and exercise were employed to reduce BMI and improve glucose responses. In the less healthy, initial state, plasma 5‐oxoproline shifted downward and required 2 h for recovery. In contrast, in the post weight‐loss state, the pattern shows nonsignificant effects. These results give further evidence of healthy subjects displaying resistance of glutathione status changes with metabolic stress. The current data indicates that when levels exceed 40 *μ*g/mg creatinine, corrections tend to require a full month, so such physiological excursions may happen only a few times annually for some individuals.

Subjects who displayed large excursions of PGA may represent people in the general population that remain largely asymptomatic while being chronically near the point of glutathione insufficiency. Thus, cases of acetaminophen‐induced high anion gap metabolic acidosis may be individuals who display such high‐variance PGA. And individuals with PGA behavior similar to the 12 low‐variance subjects may have little tendency to display such metabolic effects from usual doses of drugs that are cleared by glutathione conjugation.

## Conclusions

These data suggest that individuals may be classified by urinary PGA levels into those with higher and lower degrees of stability of glutathione status, and that individuals with metabolic stress on demands for glutathione may display 4‐week‐long patterns in PGA representing recovery of glutathione status. They also provide evidence regarding levels 15 and 40 *μ*g/mg creatinine as limits of PGA excretion that may correspond to a triggering event for shifting sulfur metabolism to normalize glutathione status. Further investigations over greater time intervals are needed to confirm the presence and characteristics of such month‐long trends of urinary PGA and how they might change with the type of challenge presented.

## Conflict of Interest

None decalred.
